# Well-Designed Bone-Seeking Radiolabeled Compounds for Diagnosis and Therapy of Bone Metastases

**DOI:** 10.1155/2015/676053

**Published:** 2015-05-14

**Authors:** Kazuma Ogawa, Atsushi Ishizaki

**Affiliations:** Division of Pharmaceutical Sciences, Graduate School of Medical Sciences, Kanazawa University, Kakuma-machi, Kanazawa 920-1192, Japan

## Abstract

Bone-seeking radiopharmaceuticals are frequently used as diagnostic agents in nuclear medicine, because they can detect bone disorders before anatomical changes occur. Furthermore, their effectiveness in the palliation of metastatic bone cancer pain has been demonstrated in the clinical setting. With the aim of developing superior bone-seeking radiopharmaceuticals, many compounds have been designed, prepared, and evaluated. Here, several well-designed bone-seeking compounds used for diagnostic and therapeutic use, having the concept of radiometal complexes conjugated to carrier molecules to bone, are reviewed.

## 1. Introduction

**Figure 1:**
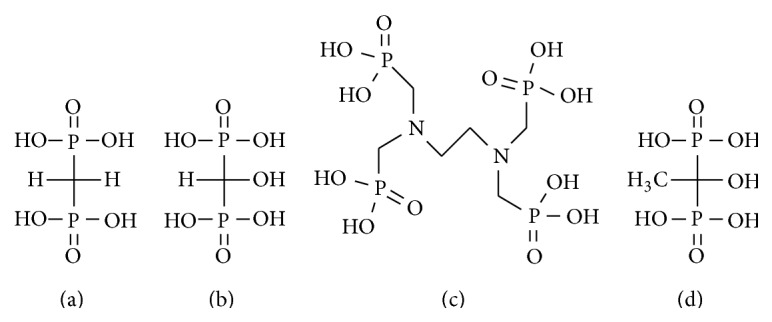
Chemical structures of bisphosphonates analogs (a) MDP, (b) HMDP, (c) EDTMP, and (d) HEDP.

For many years, ^99m^Tc-bisphosphonate complexes, ^99m^Tc-methylenediphosphonate (^99m^Tc-MDP, Figure 1(a)) and ^99m^Tc-hydroxymethylenediphosphonate (^99m^Tc-HMDP, Figure 1(b)), have been clinically used for nuclear medical imaging of metastatic bone cancer [1–4], because their high sensitivity can detect bone metastases before occurrence of anatomical changes. Bone metastases are characterized as osteolytic, osteosclerotic, or mixed type of osteolytic and osteosclerotic; namely, osteolytic or osteosclerotic changes occur in lesion sites of bone metastases. These anatomical changes could cause pathologic fractures and severe pain.

It has been known that strontium (Sr) acts as calcium mimic and accumulates in high osteoblastic activity lesions since strontium is one of the alkaline earth metals [5]. ^89^Sr has a physical half-life of 50.5 days and emits beta particles with a maximum energy of 1.46 MeV (Table 1). Strontium-89 chloride (^89^SrCl_2_, Metastron) was the first radiopharmaceutical approved for the palliation of metastatic bone pain by the US Food and Drug Administration (FDA). ^89^SrCl_2_ for the palliation of metastatic bone pain for breast cancer patients and prostate cancer patients showed a pain relief rate of 57–92%. These studies are summarized in reviews [6–9].

Samarium-153 (^153^Sm) has a physical half-life of 46.3 hours and emits beta particles with a maximum energy of 0.81 MeV (20%), 0.71 MeV (49%), and 0.64 MeV (30%) and a 28% abundance of gamma rays with energy of 103 keV (Table 1). ^153^Sm-ethylenediaminetetramethylene phosphonic acid (EDTMP, Quadramet) is a complex of ^153^Sm and EDTMP (Figure 1(c)), which has high affinity for bone mineral. ^153^Sm-EDTMP was approved and has been widely used in the United States for palliation of metastatic bone pain. The biodistribution of ^153^Sm-EDTMP is similar to that of bone scintigraphic agents such as ^99m^Tc-MDP (methylene diphosphonate) [10]. Accordingly, it was reported that the dosimetry of ^153^Sm-EDTMP could be predicted using ^99m^Tc-MDP bone scintigraphy [11]. ^153^Sm-EDTMP showed a pain relief rate in 62–84% of patients with metastatic bone pain. These studies are also summarized in reviews [6–8]. Meanwhile, in a study of comparison between the effects of the ^89^SrCl_2_ and ^153^Sm-EDTMP to patients with bone metastases, there was no statistical difference in response rates [12].

Zoledronic acid (Zometa), which is a bisphosphonate compound, has been widely used for the prevention of skeletal complications. Lam et al. combined zoledronic acid and ^153^Sm-EDTMP to treat hormone-refractory prostate cancer patients [13]. It was concluded that zoledronic acid treatment does not influence ^153^Sm-EDTMP skeletal uptake and combined treatment is feasible and safe.

The therapeutic bone-seeking radiopharmaceutical radium-223 chloride (^223^RaCl_2_) was approved by FDA and European Medicines Agency (EMA) in 2013 based on data from a phase III randomized trial (the Alpharadin in Symptomatic Prostate Cancer Patients: ALSYMPCA). Surprisingly, ^223^RaCl_2_ significantly improved overall survival in patients with castration-resistant prostate cancer with bone metastases in the ALSYMPCA study [14, 15]. In addition, because it is the first radiopharmaceutical emitting alpha particles approved for clinical use, ^223^RaCl_2_ is currently attracting much attention.

^99m^Tc-MDP, ^99m^Tc-HMDP, ^89^SrCl_2_, ^153^Sm-EDTMP, and ^223^RaCl_2_ are milestones in the development of bone-seeking radiopharmaceuticals for clinical use (Table 2). Although developing superior bone-seeking compounds is difficult, we reviewed the promising well-designed bone-seeking compounds for diagnosis and therapy of bone metastases in basic research.

## 2. ^99m^Tc-Complex-Conjugated Bisphosphonate Compounds Designed to Overcome Drawbacks of ^99m^Tc-MDP and ^99m^Tc-HMDP Complexes

Although ^99m^Tc-MDP and ^99m^Tc-HMDP are considered to be the gold standards for bone scintigraphy agents, they have not yet been optimized from a chemical and pharmaceutical perspective, because these complexes are not well-defined single-chemical species but are mixtures of short-chain and long-chain oligomers [16]. Moreover, the phosphonate groups in ^99m^Tc-MDP and ^99m^Tc-HMDP are used both as ligands for coordination and as carriers to hydroxyapatite (HA) in bone [17], which may decrease the inherent affinity of MDP and HMDP for bone. To overcome these drawbacks, a more logical design strategy has been proposed on the basis of the conjugation of a stable radiometal complex to a carrier molecule to bone. This drug design allows the ligand and carrier function to work independently and effectively.

**Figure 2:**
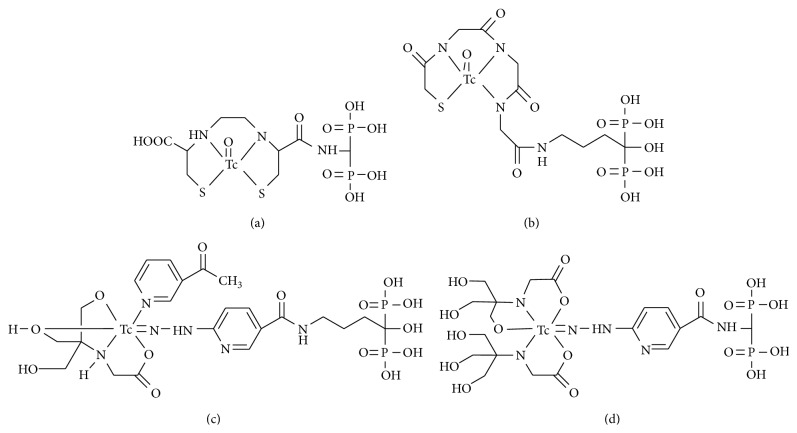
Chemical structures of ^99m^Tc-complex-conjugated bisphosphonate compounds: (a) ^99m^Tc-EC-AMDP, (b) ^99m^Tc-MAG3-HBP, (c) ^99m^Tc-HYNIC-HBP, and (d) ^99m^Tc-HYNIC-AMDP.

In 2002, Verbeke et al. described ^99m^Tc-l,l-ethylenedicysteine (EC) complex, a renal tracer agent known to have rapid renal excretion, conjugated to bisphosphonate (^99m^Tc-EC-AMDP, Figure 2(a)) [18]. ^99m^Tc-EC-AMDP showed faster blood clearance and a higher bone/blood ratio compared with ^99m^Tc-MDP in animal experiments.

In 2006, we reported ^99m^Tc-mercaptoacetylglycylglycylglycine- (MAG3-) conjugated bisphosphonate (^99m^Tc-MAG3-HBP, Figure 2(b)) and ^99m^Tc-6-hydrazinonicotinic acid (HYNIC) with tricine and 3-acetylpyridine as coligands conjugated to bisphosphonate (^99m^Tc-HYNIC-HBP, Figure 2(c)) [19]. In* in vitro* HA binding experiments, the binding rates of ^99m^Tc-MAG3-HBP and ^99m^Tc-HYNIC-HBP to HA were higher than those of ^99m^Tc-HMDP. In a biodistribution study in rats, ^99m^Tc-MAG3-HBP and ^99m^Tc-HYNIC-HBP showed higher accumulation in bone compared with ^99m^Tc-HMDP reflecting the* in vitro* findings. The blood clearance of ^99m^Tc-MAG3-HBP was delayed because of the high rate of protein binding in blood and the bone/blood ratio of ^99m^Tc-MAG3-HBP was lower than that of ^99m^Tc-HMDP. In contrast, the blood clearance of ^99m^Tc-HYNIC-HBP was as rapid as that of ^99m^Tc-HMDP and its bone/blood ratio was higher.

Liu et al. reported findings on ^99m^Tc-HYNIC-conjugated bisphosphonate (^99m^Tc-HYNIC-AMDP, Figure 2(d)) in 2011 [20]. The authors found that ^99m^Tc-HYNIC-AMDP had a higher bone uptake and higher bone/blood and bone/muscle ratios at an early time point after injection as compared with ^99m^Tc-MDP. In that study, ^99m^Tc-HYNIC-AMDP showed favorable biodistribution as a bone-seeking agent, but the bone accumulation of ^99m^Tc-MDP, a bone scintigraphy agent as a control, appeared to be too low. Two tricine molecules are used as coligands in ^99m^Tc-HYNIC-AMDP. However, as it has been reported, the complex [^99m^Tc](HYNIC)(tricine)_2_ is not stable and exists in multiple forms, and the pharmacokinetics could be affected by the exchange reaction between tricine and protein in the plasma and tissues [21–23]. The pharmacokinetics of ^99m^Tc-HYNIC-AMDP may be improved by exchanging one tricine molecule for another molecule, such as one of the pyridine derivatives.

**Figure 3:**
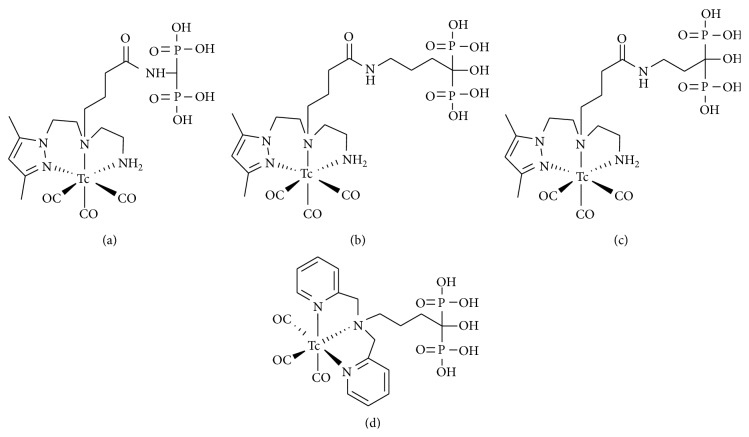
Chemical structures of ^99m^Tc-tricarbonyl-complex-conjugated bisphosphonate compounds: (a) [^99m^Tc (CO)_3_(PzNN-BP)], (b) [^99m^Tc (CO)_3_(PzNN-ALN)], (c) [^99m^Tc (CO)_3_(PzNN-PAM)], and (d) ^99m^Tc (CO)_3_-DPA-alendronate.

Palma et al. described ^99m^Tc-tricarbonyl complex, which is anchored by a pyrazolyl- (Pz-) containing ligand, conjugated to bisphosphonate compounds ([^99m^Tc(CO)_3_(PzNN-BP)], [^99m^Tc(CO)_3_(PzNN-ALN)], and [^99m^Tc(CO)_3_(PzNN-PAM)], Figures 3(a)–3(c)) [24, 25]. The structures of these technetium complexes were confirmed by reversed phase (RP) HPLC analyses. The identical retention time as the corresponding nonradioactive rhenium (Re) complexes revealed the structural analogy. Although [^99m^Tc(CO)_3_(PzNN-BP)] showed moderate bone uptake, the uptake was lower than that of ^99m^Tc-MDP. In contrast, the bone accumulation of [^99m^Tc(CO)_3_(PzNN-ALN)] and [^99m^Tc(CO)_3_(PzNN-PAM)] was high and comparable to that of ^99m^Tc-MDP. The bone/blood and bone/muscle ratios of [^99m^Tc(CO)_3_(PzNN-ALN)] and [^99m^Tc(CO)_3_(PzNN-PAM)] were higher than that of ^99m^Tc-MDP at 4 hours after injection because of their fast clearance. The difference in bone accumulation among ^99m^Tc-tricarbonyl complex-conjugated bisphosphonate compounds could be derived from the introduction of a hydroxyl group at the central carbon of the bisphosphonate because bisphosphonates containing the hydroxyl group have been reported to have higher affinity for bone minerals [26–28].

In 2009, De Rosales et al. described ^99m^Tc-tricarbonyl complex-conjugated bisphosphonate that has the structure similar to that of [^99m^Tc(CO)_3_(PzNN-ALN)] but with dipicolylamine (DPA) was used as a chelation site (^99m^Tc(CO)_3_-DPA-alendronate, Figure 3(d)) [29].* In vitro*  study showed that ^99m^Tc(CO)_3_-DPA-alendronate had higher affinity for HA than ^99m^Tc-MDP. In animal experiments, ^99m^Tc(CO)_3_-DPA-alendronate showed high uptake in bone, comparable to ^99m^Tc-MDP.

As mentioned above, certain ^99m^Tc-complex-conjugated bisphosphonate compounds have shown favorable biodistribution as bone imaging agents and higher bone/blood ratios compared with those of ^99m^Tc-MDP or ^99m^Tc-HMDP. Consequently, these results suggest that the strategy of developing stable ^99m^Tc-complex-conjugated bisphosphonates is promising.

## 3. Radiogallium-Complex-Conjugated Bisphosphonate Compounds as Bone Imaging Agents for Positron Emission Tomography (PET)

^68^Ga is a practical and interesting radionuclide for clinical PET because of its radiophysical properties, particularly as a ^68^Ge/^68^Ga generator-produced radionuclide has a half-life (*T*_1/2_) of 68 minutes (Table 1) [30]. It does not require an on-site cyclotron and can be eluted on demand. Indeed, in principle, the long half-life of the parent nuclide ^68^Ge (*T*_1/2_ = 270.8 days) provides a generator with a long lifespan.

The above-mentioned drug design concept, which is a stable complex-conjugated bisphosphonate, could also be applicable to gallium complexes. With the aim of developing a superior bone imaging PET tracer, some types of radiogallium complex-conjugated bisphosphonate compounds have been reported.

**Figure 4:**
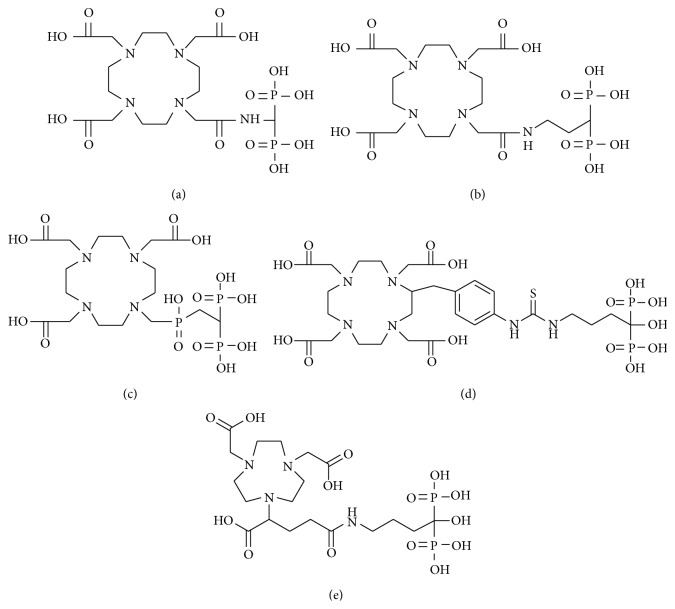
Chemical structures of precursors of ^67/68^Ga complex-conjugated bisphosphonate compounds: (a) BPAMD, (b) BPAPD, (c) BPPED, (d) DOTA-Bn-SCN-HBP, and (e) NOTA-BP.

**Figure 5:**
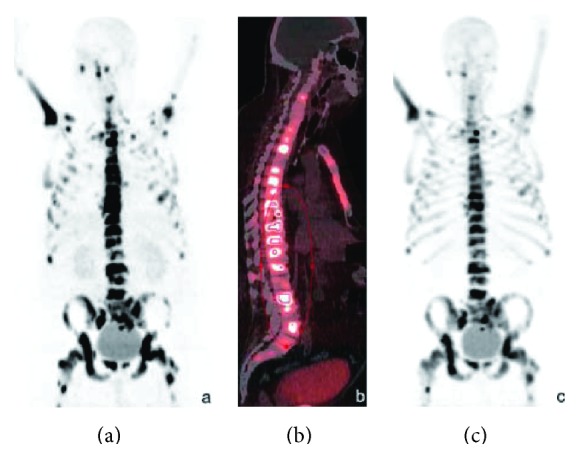
^68^Ga-BPAMD was injected intravenously into a patient with extensive bone metastases of prostate cancer. ^68^Ga-BPAMD [maximum intensity projection (MIP) 50 min after injection (p.i.), 462 MBq] revealed intense accumulation in multiple osteoblastic lesions in the central skeleton, ribs, and proximal extremities: (a) = coronal PET, (b) = sagittal PET/CT. For comparison, (c) shows ^18^F-fluoride PET (sagittal, MIP 90 min p.i., 270 MBq). With kind permission from Springer Science+Business Media: Eur J Nucl Med Mol Imaging, PET/CT imaging of osteoblastic bone metastases with ^68^Ga-bisphosphonates: first human study, 37, 2010, 834, Fellner et al.

In 2010, Fellner et al. reported a human study of ^68^Ga-DOTA-conjugated bisphosphonate (^68^Ga-BPAMD, Figure 4(a)), containing 1,4,7,10-tetraazacyclododecane-1,4,7,10-tetraacetic acid (DOTA) as a ligand for gallium [31]. ^68^Ga-BPAMD showed high uptake in osteoblastic metastases lesions in a patient with prostate cancer (Figure 5). The maximal standardized uptake value (SUV_max⁡_) was 77.1 and 62.1 in the 10th thoracic and L2 vertebra for ^68^Ga-BPAMD compared with respective values of 39.1 and 39.2 for ^18^F-fluoride, which is a typical bone imaging agent for PET (Table 2), respectively. In 2012, Fellner et al. reported the findings of basic experiments on ^68^Ga-BPAMD using *μ*-PET with bone metastasis rat model [32]. ^68^Ga-BPAMD highly accumulated in metastatic bone lesions compared with healthy bone in the same animal (contrast factor = 3.97 ± 1.82). The same research group further reported ^68^Ga-DOTA-conjugated bisphosphonate derivatives, ^68^Ga-BPAPD and ^68^Ga-BPPED (Figures 4(b) and 4(c)) in 2013 [33]. The phosphinate-conjugated bisphosphonate ^68^Ga-BPPED showed higher accumulation in bone compared with ^68^Ga-BPAMD and ^68^Ga-BPAPD, amide-conjugated bisphosphonates. The presence of phosphinate may contribute to an additional binding to HA, leading to higher accumulation in bone.

In 2011, we also reported ^67^Ga-DOTA complex-conjugated bisphosphonate (^67^Ga-DOTA-Bn-SCN-HBP, Figure 4(d)) [34]. Although the aim was to develop a superior ^68^Ga-labeled bone imaging agent for PET, in the initial basic studies ^67^Ga was used because of its longer half-life. In biodistribution experiments in normal mice, ^67^Ga-DOTA-Bn-SCN-HBP rapidly and highly accumulated in bone but was rarely observed in tissues other than bone. As a result, the bone/blood ratio of ^67^Ga-DOTA-Bn-SCN-HBP was comparable to that of ^99m^Tc-HMDP, which is a gold standard for a bone scintigraphy agent.

Furthermore, in 2011, Suzuki et al. reported ^68^Ga-NOTA-conjugated bisphosphonate, containing 1,4,7-triazacyclononane-1,4,7-triacetic acid (NOTA) as a ligand for gallium (^68^Ga-NOTA-BP, Figure 4(e)) [35]. In biodistribution experiments using Wistar rats, ^68^Ga-NOTA-BP showed faster clearance and a higher bone/blood ratio than ^99m^Tc-MDP and ^18^F-fluoride. Moreover, in PET study using a mouse model of bone metastasis, ^68^Ga-NOTA-BP showed high accumulation of radioactivity in osteolytic lesions in the tibia.

These results suggest that the drug design concept of radio gallium complex-conjugated bisphosphonate could be useful for the development of ^68^Ga PET imaging agents for bone disorders such as bone metastases.

## 4. Re-Complex-Conjugated Bisphosphonate for Palliation of Bone Metastases

Gamma ray emitter radionuclide and positron emitter radionuclide-labeled bone-seeking agents are used for the diagnosis of bone metastases. A prominent symptom caused by bone metastases is pain, which has a significant impact on the patients' quality of life. Bone-seeking agents labeled with high-energy beta particle emitter radionuclides and alpha particle emitter radionuclides are used for palliation of pain caused by bone metastases. Rhenium, which has similar chemical properties to technetium, because they are members of family VIIA of the periodic table, has two useful radionuclides, ^186^Re and ^188^Re, which are useful for radionuclide therapy [36]. Both rhenium radionuclides emit not only beta particles for therapy but also gamma rays, which are suitable for diagnoses: ^186^Re (*T*_1/2_ = 3.78 days, *β*_max⁡_^−^ = 1.07 MeV, *γ* = 137 keV) and ^188^Re (*T*_1/2_ = 16.98 hours, *β*_max⁡_^−^ = 2.12 MeV, *γ* = 155 keV) (Table 1). In addition, ^188^Re has a further advantage for clinical use because it is obtained from an in-house alumina-based ^188^W/^188^Re generator, similar to a ^99^Mo/^99m^Tc generator [37].

When considering the use of rhenium in bone-seeking agents for palliation, similar to ^99m^Tc-MDP and ^99m^Tc-HMDP, it is known that rhenium coordinates with some bisphosphonate derivatives. ^186/188^Re-1-hydroxyethylidene-1,1-diphosphonate (^186/188^Re-HEDP, Figure 1(d)), which has high affinity for bone, has been prepared and used for clinical research [9, 38, 39]. Although the chemical properties of rhenium are similar to those of technetium as mentioned above, rhenium is more easily oxidized than technetium [40], and it has been reported that ^186^Re-HEDP is not as stable as ^99m^Tc-bisphosphonate complexes [41]. Some studies reported that ^186^Re-HEDP showed unexpected gastric uptake in patients with bone metastases [42, 43]. This may be derived from the accumulation of free rhenium (perrhenate: ReO_4_^−^) in the stomach due to the instability of ^186^Re-HEDP [40, 44]. Moreover, as with ^99m^Tc-MDP and ^99m^Tc-HMDP, the phosphonate groups in ^186/188^Re-HEDP are used as both ligands for coordination and as carrier to HA in bone, which may decrease the inherent affinity of HEDP for bone.

**Figure 6:**
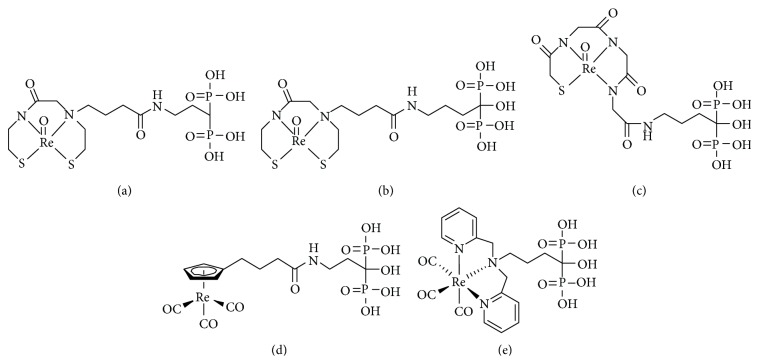
Chemical structures of ^186/188^Re-complex-conjugated bisphosphonate compounds: (a) ^186^Re-MAMA-BP, (b) ^186^Re-MAMA-HBP, (c) ^186^Re-MAG3-HBP, (d) [^186^Re]CpTR-Gly-APD, and (e) ^188^Re(CO)_3_-DPA-alendronate.

To overcome these problems, designing a stable ^186/188^Re-complex-conjugated bisphosphonate would be useful. Therefore, we studied ^186^Re-monoaminemonoamidedithiol- (MAMA-) and ^186^Re-mercaptoacetylglycylglycylglycine- (MAG3-) conjugated bisphosphonate compounds (^186^Re-MAMA-BP, ^186^Re-MAMA-HBP, and ^186^Re-MAG3-HBP: Figures 6(a)–6(c)) and reported their findings in 2004, 2006, and 2005, respectively [28, 45, 46]. In an* in vitro* stability experiment in buffer solution, the Re-complex-conjugated bisphosphonate compounds, ^186^Re-MAMA-HBP, ^186^Re-MAMA-BP, and ^186^Re-MAG3-HBP, were more stable than ^186^Re-HEDP. In the biodistribution experiments performed in normal mice, ^186^Re-MAMA-HBP, ^186^Re-MAMA-BP, and ^186^Re-MAG3-HBP showed lower accumulation of radioactivity in stomach compared with ^186^Re-HEDP. This result indicates that the drug design of Re-complex-conjugated bisphosphonates enabled better stability* in vitro* and* in vivo*. Of ^186^Re-complex-conjugated bisphosphonate compounds, ^186^Re-MAG3-HBP showed the most favorable biodistribution characteristics as bone-seeking radiopharmaceuticals such as high and selective bone accumulation, based on high hydrophilicity (log *P* value: −2.68 ± 0.01) and the introduction of a hydroxyl group to the central carbon of the bisphosphonate structure. Previous studies suggested that the hydroxyl group affects affinity for bone minerals [26, 27]. We evaluated the therapeutic potential of ^186^Re-MAG3-HBP for the palliation of metastatic bone pain using an animal model of bone metastasis [47]. The planar image of ^186^Re-MAG3-HBP showed high accumulation of radioactivity in bone metastasis lesion. ^186^Re-MAG3-HBP was more effective for palliation and was compared with ^186^Re-HEDP using the hind paw withdrawal response to stimulation with von Frey filaments. Moreover, although ^186^Re-HEDP did not affect tumor growth, ^186^Re-MAG3-HBP significantly inhibited tumor growth.

In 2007, Uehara et al. reported [^186^Re]CpTR-Gly-APD (Figure 6(d)), which is a tricarbonyl [^186^Re][(cyclopentadienylcarbonyl amino)-acetic acid] rhenium complex ([^186^Re]CpTR-Gly)-conjugated bisphosphonate [48]. [^186^Re]CpTR-Gly-APD showed characteristics superior to those of ^186^Re-HEDP, such as higher stability in plasma, a higher binding rate for HA, higher bone accumulation, and lower plasma protein binding. When [^186^Re]CpTR-Gly-APD with HEDP (9.0 mg/kg) was administered to mice, the accumulation of radioactivity in bone significantly decreased and the blood clearance was delayed. Therefore, the authors concluded that the specific activity of ^186^Re-labeled bisphosphonate compounds is very important to bone accumulation and blood clearance.

In the ^99m^Tc-complex-conjugated bisphosphonate session, ^99m^Tc(CO)_3_-DPA-alendronate has been discussed above. Using the same ligand, in 2010, De Rosales et al. reported ^188^Re(CO)_3_-DPA-alendronate (Figure 6(e)) [49]. ^188^Re(CO)_3_-DPA-alendronate can easily be synthesized with high specific activities and high yields (≥96%). ^188^Re(CO)_3_-DPA-alendronate showed higher stability* in vitro* compared with ^188^Re-HEDP, which oxidized to ^188^ReO_4_^−^ (up to 75%) when placed in PBS for 48 hours at 37°C. In* in vivo* imaging, ^188^Re(CO)_3_-DPA-alendronate showed superior biodistribution of radioactivity than ^188^Re-HEDP; that is, ^188^Re(CO)_3_-DPA-alendronate highly accumulated in metabolically active bone, such as the joints with low soft-tissue uptake.

These results indicate that the concept of the stable ^186^Re-complex-conjugated bisphosphonates could be more useful and that novel ^186^Re-complex-conjugated bisphosphonate complexes could be attractive candidates as palliative agents in metastatic bone pain.

## 5. Aspartic Acid Peptides as Carriers of Radionuclides to Bone

Several major noncollagenous bone proteins, such as osteopontin and bone sialoprotein, have repeating sequences of acidic amino acids (Asp or Glu) in their structures, offering potential HA binding sites [50–52]. It has been reported that polyglutamic and polyaspartic acids have a high affinity for HA and could be used as carriers for drug delivery to bone [53–55].

**Figure 7:**
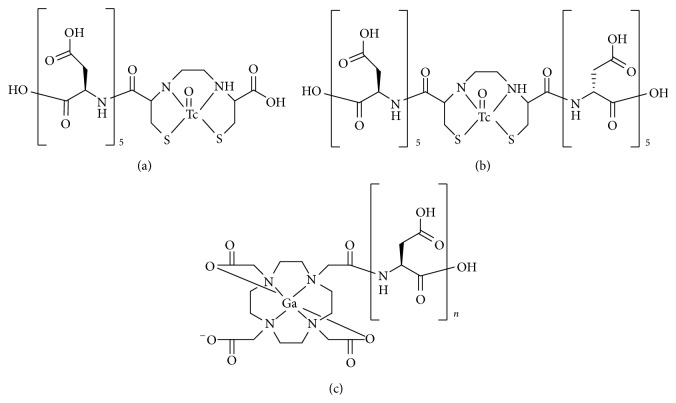
Chemical structures of radiometal complex-conjugated aspartic acid peptide compounds: (a) ^99m^Tc-EC-(d-Asp)_5_, (b) ^99m^Tc-EC-[(d-Asp)_5_]_2_, and (c) ^67^Ga-DOTA-(Asp)_*n*_.

In 2013, Yanagi et al. reported ^99m^Tc-complex-conjugated aspartic acid (Asp) peptides [56]. They selected EC as a ligand to prepare stable technetium complexes, conjugated with one or two penta d-Asp peptides [^99m^Tc-EC-(d-Asp)_5_ or ^99m^Tc-EC-[(d-Asp)_5_]_2_, Figures 7(a) and 7(b)]. The HA binding of ^99m^Tc-EC-[(d-Asp)_5_]_2_ was higher than that of ^99m^Tc-EC-(d-Asp)_5_. ^99m^Tc-EC-[(d-Asp)_5_]_2_ showed significantly lower accumulation in normal bone of mice compared with ^99m^Tc-MDP. In contrast, when compared with ^99m^Tc-MDP, ^99m^Tc-EC-[(d-Asp)_5_]_2_ showed the same degree of accumulation in a osteogenic lesion of tumor-bearing rat models. Thus, the uptake ratio of osteogenic lesion to normal bone (osteogenic lesion/normal bone) of ^99m^Tc-EC-[(d-Asp)_5_]_2_ after injection was higher than that of ^99m^Tc-MDP. The authors supposed that the higher osteogenic lesion/normal bone ratio derived forms the higher molecular size, which was determined by permeability through a membrane filter (10 kDa), of ^99m^Tc-EC-[(d-Asp)_5_]_2_ compared with that of ^99m^Tc-MDP and ^99m^Tc-EC-(d-Asp)_5_.

In 2013, we reported ^67^Ga-DOTA-conjugated l-Asp peptides (^67^Ga-DOTA-(Asp)_*n*_, Figure 7(c)), which had varying peptide lengths (*n* = 2, 5, 8, 11, or 14) [57]. Binding affinity to HA of ^67^Ga-DOTA-(Asp)_*n*_ increased with an increase in the length of the aspartate peptide. The HA binding of ^67^Ga-DOTA-conjugated bisphosphonate, ^67^Ga-DOTA-Bn-SCN-HBP, was inhibited by lower concentrations of alendronate, one of bisphosphonate compounds, compared with ^67^Ga-DOTA-(Asp)_14_. In biodistribution experiments of normal mice, ^67^Ga-DOTA-(Asp)_8_, ^67^Ga-DOTA-(Asp)_11_, and ^67^Ga-DOTA-(Asp)_14_ selectively and highly accumulated in bone (10.5 ± 1.5, 15.1 ± 2.6, and 12.8 ± 1.7% ID/g, resp.). Although the bone accumulation of ^67^Ga-DOTA-(Asp)_*n*_ was lower than that of ^67^Ga-DOTA-Bn-SCN-HBP, the blood clearance of ^67^Ga-DOTA-(Asp)_*n*_ was more rapid. Accordingly, the bone/blood ratios of ^67^Ga-DOTA-(Asp)_11_ and ^67^Ga-DOTA-(Asp)_14_ were comparable to that of ^67^Ga-DOTA-Bn-SCN-HBP. Moreover, the inhibition of radioactive bone accumulation by alendronate was greater after injection of ^67^Ga-DOTA-Bn-SCN-HBP than that of ^67^Ga-DOTA-(Asp)_14_.

These results indicate that not only bisphosphonate molecules but also acidic amino acid peptide sequences could be useful as carriers of radionuclides to bone metastases lesions. Moreover, radiometal complex-conjugated acidic amino acid peptides may provide slightly different information from radiometal complex-conjugated bisphosphonates.

## 6. Carbon-11 Labeled Cathepsin K Inhibitors

Cathepsin K is a member of the papain family of cysteine peptidases with a primary physiological function of cleavage of type I and type II collagen [58]. The enzyme is highly expressed in activated osteoclasts, and a change in the number of the osteoclast is related to bone diseases such as osteoporosis [59]. Therefore, it could be useful to determine the changes in osteoclast numbers in such disease states by imaging cathepsin K. Because many inhibitors of cathepsin K have been synthesized and evaluated both* in vitro* and* in vivo*, their derivatives may be candidates as mother compounds for cathepsin K imaging agents. The possibility of targeting cathepsin K in* in vivo* imaging, using a near-infrared reporter probe, was confirmed in a previous report [60].

**Figure 8:**
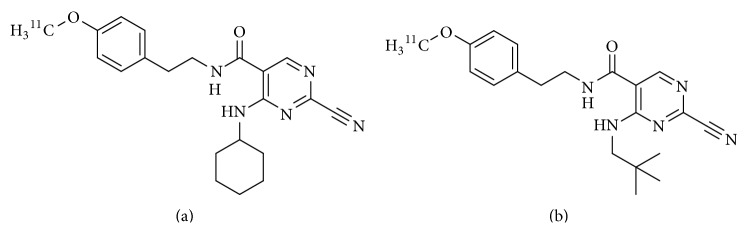
Chemical structures of carbon-11 labeled cathepsin K inhibitors: (a) 2-cyano-4-(cyclohexylamino)-N-(4-[^11^C]methoxyphenethyl)-pyrimidine-5-carboxamide and (b) 2-cyano-N-(4-[^11^C]methoxyphenethyl)-4-(neopentylamino) pyrimidine-5-carboxamide.

Rodnick et al. reported carbon-11-labeled cathepsin K inhibitors with high affinity as cathepsin K imaging agents, 2-cyano-4-(cyclohexylamino)-N-(4-[^11^C]methoxyphenethyl)-pyrimidine-5-carboxamide ([^11^C]**1**, Figure 8(a)) and 2-cyano-N-(4-[^11^C]methoxyphenethyl)-4-(neopentylamino) pyrimidine-5-carboxamide ([^11^C]**2**, Figure 8(b)) in 2014 [61]. Nonradioactive counterparts of [^11^C]**1** and [^11^C]**2** were reported in 2007 [62]. In that study, because the pyrimidine core structure docked into the cathepsin K active site, many types of derivatives based on a pyrimidine scaffold were synthesized and evaluated as cathepsin K inhibitors. Among them, the nonradioactive counterparts showed greater affinity and selectivity for cathepsin K. For inhibition of cathepsin K, cathepsin L, and cathepsin S, IC_50_ values of compound** 1** were 0.022, 0.17, and 0.7, and those of compound** 2** were <0.003, 1.2, and 0.9, respectively. [^11^C]**1** and [^11^C]**2** were radiosynthesized by standard reaction conditions used for alkylation reactions with [^11^C]methyl iodide.* In vivoμ*-PET imaging experiments showed that [^11^C]**1** and [^11^C]**2** inhibitors have a higher uptake in actively growing bone regions, such as distal ulnar, carpal, distal and proximal humeral, distal femur, and proximal tibia, than in nontarget regions such as muscle. The uptake in specific bone regions was based on specific binding to cathepsin K because the uptake was inhibited by pre- or coinjection of an excess amount of ligands. These results indicated that radiolabeled cathepsin K inhibitors could have potential as* in vivo* imaging agents to determine a change in the number of osteoclasts.

## 7. Dual-Modality Single Photon Emission Computed Tomography/Near-Infrared (SPECT/NIR) Fluorescent Probe

Recently, multimodality molecular imaging combining several imaging techniques has attracted much attention in basic scientific and clinical research. Nuclear medical imaging can detect deep tissues in the body with high sensitivity, but there are some problems such as relatively poor spatial resolution [63].

**Figure 9:**
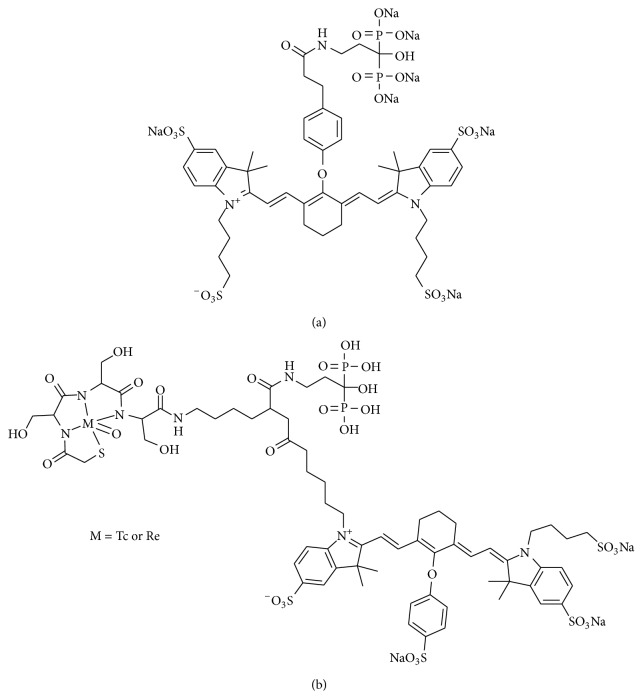
Chemical structures of a heptamethine indocyanine-type NIR fluorophore conjugated bisphosphonate: (a) Pam78 and a trifunctional diagnostic agent and (b) Pam-Tc/Re-800.

Optical imaging is a relatively new imaging modality that offers real-time and nonradioactive and high-resolution imaging of fluorophores in lesion sites, but it is difficult to detect a deep tissue using this technique [64]. Fluorescence imaging with near-infrared (NIR, 700–900 nm wavelength) light reveals relatively low tissue absorption. IRDye78 is a heptamethine indocyanine-type NIR fluorophore with peak absorption at 771 nm and peak excitation emission at 806 nm. Pam78 (Figure 9(a)), a IRDye78-conjugated pamidronate (one of the bisphosphonate derivatives), has been reported as a NIR fluorescence imaging probe targeted to HA [65]. HA is considered to be a good marker for some diseases because calcification (HA deposition) occurs during the processes of cancer and atherosclerosis.

Bhushan et al. reported the trifunctional diagnostic agent Pam-Tc/Re-800 (Figure 9(b)) in 2008 [66]. Pam-Tc/Re-800 possesses a radiometal complex as a nuclear imaging probe, a fluorescent site as a fluorescence imaging probe, and bisphosphonate having high affinity for HA as a carrier to bone in a molecule. In an* in vitro* experiment, Pam-Tc/Re-800 showed specific and selective binding to HA. In the fluorescence imaging of microcalcified breast cancer rat model, Pam-Re-800 detected breast cancer microcalcifications. In SPECT/CT imaging, Pam-Tc-800 showed not only accumulation in normal bone but also highly sensitive detection of breast cancer microcalcifications. In biodistribution experiments, the total body clearance of Pam-Tc-800 at 4 hours after injection was comparable to that of ^99m^Tc-MDP. Moreover, Pam-Tc-800 showed a higher uptake in bone and tumor than ^99m^Tc-MDP. These results indicated that the novel trifunctional agent could provide simultaneous imaging by SPECT and NIR fluorescence. Dual-modality imaging may compensate for the drawbacks of the other modalities.

## 8. Summary

In this paper, several well-designed bone-seeking compounds were reviewed. They are chemically well-characterized and different from ^99m^Tc-MDP and ^99m^Tc-HMDP. Some demonstrated superior biodistribution characteristics. The mechanism by which all the compounds (except the carbon-11 labeled cathepsin K inhibitors) accumulate in bone is derived due to a high affinity for HA. We estimate that ^68^Ga-DOTA-conjugated bisphosphonate compounds, such as ^68^Ga-BPAMD and ^68^Ga-DOTA-Bn-SCN-HBP, are the most promising diagnostic agents for bone metastases because they show superior biodistribution characteristic and ^68^Ga is a useful PET radionuclide in clinical. Moreover, as DOTA ligand could form a complex with not only ^67/68^Ga but also ^177^Lu and ^90^Y, the palliation therapy is applicable using the same ligand. Namely, this system is “theranostics”, which is a combination of diagnosis and therapy.

Thus, the information from imaging data and the type of bone metastasis susceptible to treatment should be similar to those for existing bone-seeking radiopharmaceuticals. We hope that novel bone-seeking compounds that possess a different accumulation mechanism will be developed in the near future.

## Figures and Tables

**Table 1:** Properties of radionuclides.

Radionuclide	Half-life	Maximum energy of beta particles (MeV)	Main energy of gamma ray (keV, %)	Maximum range (mm)	Main production
Tc-99m	6.02 h	None	141 (89%)	—	^ 98^Mo(n, *γ*)^99^Mo ^99^Mo/^99m^Tc generator
Ga-68	67.6 m	None	1077 (3%) 1899 (89%)^*^	—	^ 69^Ga(p, 2n)^68^Ge ^68^Ge/^68^Ga generator
Sr-89	50.5 d	1.46	910 (0.01%)	7	^ 88^Sr(n, *γ*)^89^Sr
Sm-153	1.9 d	0.81	103 (28%)	4	^ 152^Sm(n, *γ*)^153^Sm
Re-186	3.8 d	1.07	137 (9%)	5	^ 186^W(p, n)^186^Re ^185^Re(n, *γ*)^186^Re
Re-188	17.0 h	2.12	155 (15%)	10	^ 186^W(n, *γ*)^187^W ^187^W(n, *γ*)^188^W ^188^W/^188^Re generator
Ra-223	11.4 d	7.53^†^	154 (6%), 270 (14%)	<0.1	^ 227^Ac/^227^Th generator ${}^{227}\text{Th}\overset{\alpha }{\to }{\;}^{223}{\text{Ra}}^{‡}$

^*^Positron energy.

^†^ *α* energy (Ra-223 has multiple decay to stable nuclide in which 4*α* particles are generated during each decay, resulting in high energy deposition (28.2 MeV), with 95% of the energy from the *α* emissions [67].).

^‡^Ra-223 could be produced from ^227^Ac/^227^Th generator and purified using Ac-resin to immobilize ^227^Ac and ^227^Th [33].

**Table 2:** Radiopharmaceuticals approved for bone metastases by FDA or EMA.

Radiopharmaceutical	Standard dose	Use
^ 99m^Tc-MDP	370–740 MBq	Diagnosis
^ 99m^Tc-HMDP	370–740 MBq	Diagnosis
[^18^F]NaF (IASOflu)	18.5–74 MBq	Diagnosis
^ 89^SrCl_2_ (Metastron)	148 MBq 1.5–2.2 MBq/kg	Therapy
^ 153^Sm-EDTMP (Quadramet)	37 MBq/kg	Therapy
^ 223^RaCl_2_ (Xofigo)	50 kBq/kg	Therapy
